# Considerations for an *In Vitro*, Cell-Based Testing Platform for Detection of Adverse Drug-Induced Inotropic Effects in Early Drug Development. Part 1: General Considerations for Development of Novel Testing Platforms

**DOI:** 10.3389/fphar.2019.00884

**Published:** 2019-08-09

**Authors:** Brian D. Guth, Michael Engwall, Sandy Eldridge, C. Michael Foley, Liang Guo, Gary Gintant, John Koerner, Stanley T. Parish, Jennifer B. Pierson, Alexandre J. S. Ribeiro, Tanja Zabka, Khuram W. Chaudhary, Yasunari Kanda, Brian Berridge

**Affiliations:** ^1^Department of Drug Discovery Sciences, Boehringer Ingelheim Pharma GmbH & Co KG, Biberach an der Riss, Germany; ^2^PreClinical Drug Development Platform (PCDDP), North-West University, Potchefstroom, South Africa; ^3^Safety Pharmacology and Animal Research Center, Amgen Research, Thousand Oaks, CA, United States; ^4^Division of Cancer Treatment and Diagnosis, National Cancer Institute, National Institutes of Health, Bethesda, MD, United States; ^5^Department of Integrative Pharmacology, Integrated Sciences and Technology, AbbVie, North Chicago, IL, United States; ^6^Laboratory of Investigative Toxicology, Frederick National Laboratory for Cancer Research, Leidos Biomedical Research, Inc., Frederick, MD, United States; ^7^Center for Drug Evaluation and Research, US Food and Drug Administration, Silver Spring, MD, United States; ^8^Health and Environmental Sciences Institute, Washington, DC, United States; ^9^Division of Applied Regulatory Science, Office of Clinical Pharmacology, Office of Translation Sciences, Center for Drug Evaluation and Research, US Food and Drug Administration, Silver Spring, MD, United States; ^10^Department of Safety Assessment, Genentech, South San Francisco, CA, United States; ^11^Global Safety Pharmacology, GlaxoSmithKline plc, Collegeville, PA, United States; ^12^Division of Pharmacology, National Institute of Health Sciences, Kanagawa, Japan; ^13^National Toxicology Program, National Institute of Environmental Health Sciences, Durham, NC, United States

**Keywords:** contractility, inotropic state, cardiomyocyte, stem cells, myocardium

## Abstract

Drug-induced effects on cardiac contractility can be assessed through the measurement of the maximal rate of pressure increase in the left ventricle (LVdP/dt_max_) in conscious animals, and such studies are often conducted at the late stage of preclinical drug development. Detection of such effects earlier in drug research using simpler, *in vitro* test systems would be a valuable addition to our strategies for identifying the best possible drug development candidates. Thus, testing platforms with reasonably high throughput, and affordable costs would be helpful for early screening purposes. There may also be utility for testing platforms that provide mechanistic information about how a given drug affects cardiac contractility. Finally, there could be *in vitro* testing platforms that could ultimately contribute to the regulatory safety package of a new drug. The characteristics needed for a successful cell or tissue-based testing platform for cardiac contractility will be dictated by its intended use. In this article, general considerations are presented with the intent of guiding the development of new testing platforms that will find utility in drug research and development. In the following article (part 2), specific aspects of using human-induced stem cell-derived cardiomyocytes for this purpose are addressed.

## Introduction

Cardiovascular (CV) safety liabilities of new drugs (real or presumptive) are an important source of attrition during development and may have severe consequences for patients if compounds progress to clinical testing and even market approval. The demonstration of CV safety in preclinical studies is a cornerstone of the safety pharmacology assessment performed prior to first-in-human trials. Accordingly, preclinical safety assessment is enriched for CV assessments, both morphologic and functional. Despite the proven utility and relevance of *in vivo* animal systems for testing for CV effects, some liabilities have only been recognized in patients, with their varied susceptibilities (i.e., age, gender, genetics, etc.) and co-morbidities that are poorly modeled in healthy animals. Current International Council for Harmonisation of Technical Requirements for Registration of Pharmaceuticals for Human Use (ICH) guidelines (e.g., ICHS7A) focus on the evaluation of hemodynamic effects including heart rate and systemic arterial blood pressure. Although the assessment of drug-induced inotropic effects on the myocardium is not currently a routine regulatory expectation based on preclinical safety guidelines, evaluation of potential inotropic effects *in vivo* is recognized as an essential safety endpoint in drug development (Guth et al., 2015). Given its potential impact on drug development, the detection of drug-induced effects on myocardial contractility should be detected as early as possible and therefore preferably prior to the *in vivo* testing of drug candidates. The purpose of this article is to describe the necessary characteristics of a successful *in vitro* test system for employment in the context of its positioning and use within the workflows of drug research and development.

## Definition of Myocardial (Cardiac) Contractility

Contractility is defined as the intrinsic ability of cardiac muscle fibers to shorten, independent of its loading conditions and frequency of contraction. The level of contractility of the cardiac muscle is also referred to as its *inotropic* state, while the rate of contraction can be referred to as its *chronotropic* state. Furthermore, the ability of a cardiac muscle to relax following peak contraction is referred to as its *lusitropic* state. The level of cardiac contractile state directly contributes to cardiac output and therefore the maintenance of blood perfusion throughout the body. Changes in cardiac contractility are often a response to autonomic stimulation, an adaptation to metabolic demands, or they can result from a pathological insult such as seen in congestive heart failure (Blinks and Koch-Weser, 1961; Suga et al., 1973). As surrogates for myocardial contractility (or inotropy), some hemodynamic indices are closely related to the strength of myocardial contraction, such as left ventricular pressure generation, ejection fraction, stroke volume, and arterial blood pressure, but they may involve invasive surgical catheterization (Mason et al., 1971) or echocardiography. The contractile state of the myocardium determines the extent of myocardial shortening and pump performance, such that these are often used as clinical biomarkers in its place (Mason et al., 1971).

## Clinical Relevance of Changes in Cardiac Contractility

Reductions in cardiac contractility can lead to systemic hemodynamic effects such as a drop in systemic arterial blood pressure (hypotension), an increased heart rate, syncope, and loss of blood flow to vital organs. Recent studies suggest that drug-induced orthostatic hypotension is a leading cause of syncope in patients who present with this symptom in the emergency room, and it serves as an independent risk factor for rehospitalization in patients suffering from CV disease (Sarasin et al., 2002; Onuki et al., 2017). Increases in cardiac contractility can be equally problematic clinically, leading to myocardial hypertrophy (if chronic), impaired myocardial energetics, and increased pro-arrhythmic potential. While inotropic support is a commonly used treatment for patients suffering from impaired contractile function, for the purposes of this review, our focus remains the investigation of unwanted drug-induced alterations in contractility and the identification of its associated risk in pharmaceutical research and development. Furthermore, positive inotropes prescribed to heart failure patients, including dobutamine and other catecholamines, often have small safety windows between clinical benefit and toxicity (Leier, 1986; Schwinger et al., 1993; Overgaard and Dzavik, 2008). Finally, advances in our ability to diagnose and treat disease at early stages, such as cancer, have led to increased observations of chronic, treatment-induced reductions in cardiac contractility. In a recent report, a tyrosine kinase inhibitor (sunitinib) prescribed to patients with gastrointestinal stromal tumors was associated with >15% reduction in myocardial ejection fraction and development of heart failure in roughly 18% of patients (Kerkela et al., 2009; Cheng and Force, 2010). Clearly, the careful evaluation of acute and chronic treatment-induced effects of pharmaceutical agents on cardiac contractility is important in bringing safer medicines to the clinic.

## Intracellular Mechanisms Affecting Cardiac Contractility

**Figure 1:**
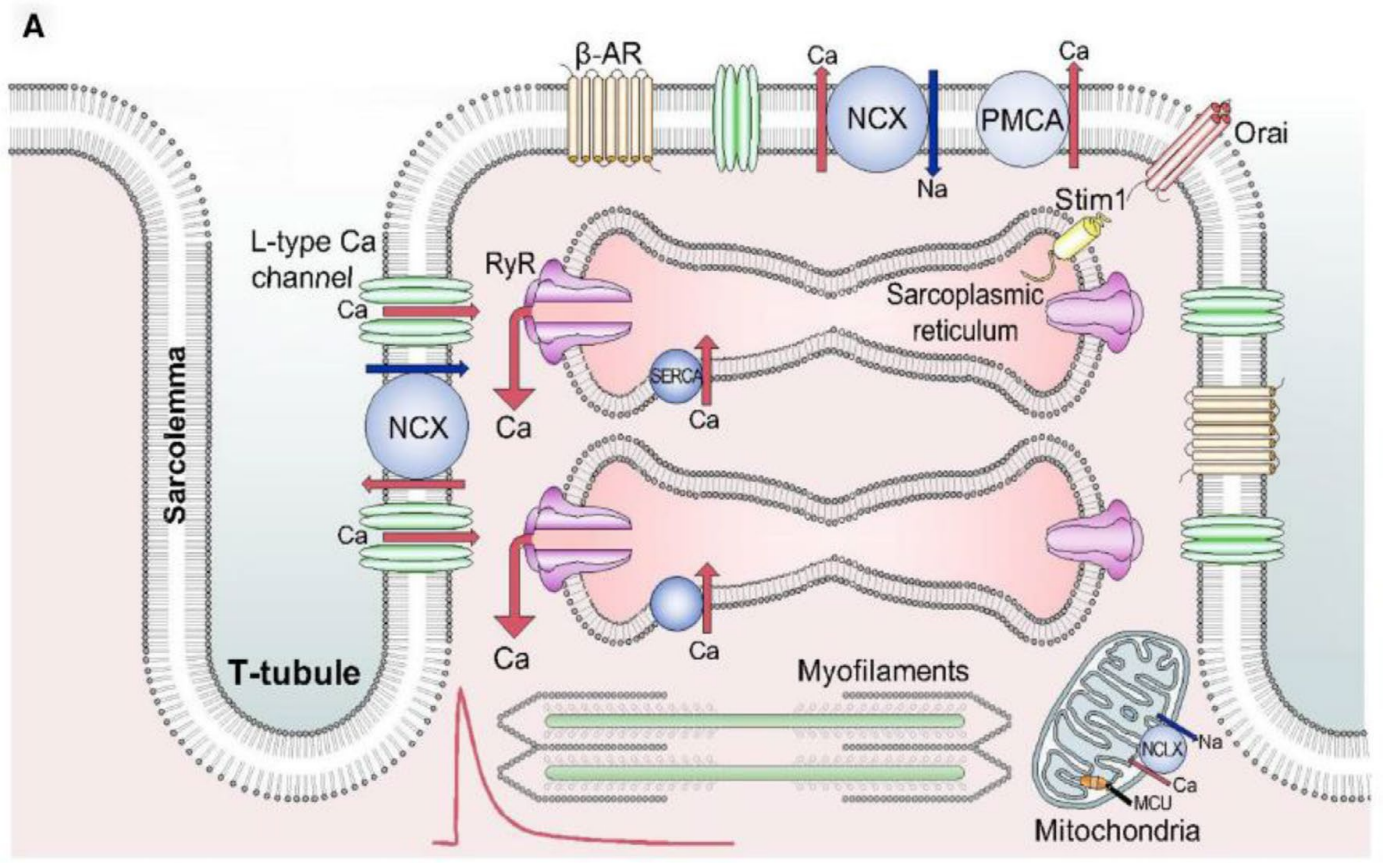
Schematic diagram of the elements playing a role in the contractile process and therefore being potential targets for drug-induced effects on myocardial contractility. From Eisner et al. (2017) with permission. NCX, sodium-calcium exchanger; PMCA, plasma membrane calcium ATPase; RyR, ryanodine receptor; MCU, mitochondrial calcium uniporter; NCLX, mitochondrial sodium-calcium exchanger.

Normal cardiac force generation is the product of several interrelated and interdependent physiological processes at the cellular and organ levels (**Figure 1**). Propagating action potentials originating at the sinoatrial node have a generation rate that is modulated by the autonomic nervous system. These action potentials at the cellular level activate transmembrane ionic currents that include influx of calcium ions. The resultant elevation of intracellular-free calcium (calcium transient) induces the release of internal calcium stores from the sarcoplasmic reticulum that, in turn, leads to the release of inhibitory tropomyosin complexes from interdigitating actin and myosin myofibrils, allowing them to slide along their long axis to elicit cell shortening. Active, ATP-dependent reuptake of intracellular calcium into the sarcoplasmic reticulum by energy-dependent SERCA leads to cell re-lengthening and a state of relaxation.

The overall force of contraction of cardiac muscle or isolated cardiac myocytes is determined by both its inotropic state and the length-tension relationship of the cardiac muscle (Frank–Starling mechanism). Cardiac contractility (inotropy) specifically refers to the force generation for a given length and therefore independent of length-related changes in force of contraction. Most cellular mechanisms that affect inotropic state involve changes in Ca^++^ handling or in Ca^++^ sensitivity of the contractile proteins that are largely length-independent. Integrated experimental models to assess changes in inotropy independent of influences of length-tension, heart rate/beating rate, afterload, and autonomic influences are challenging to interpret, as these various factors influence force generation.

## Detection of Drug-Induced Effects on Cardiac Contractility

Drug-induced effects on this complex system can be detected using experimental models in which the contraction of an entire heart (either *ex vivo* or *in situ*), ( McVeigh et al., 1998; Liao et al., 2012; Wallis et al., 2015; Jan and Tajik, 2017), or extracted portions thereof [e.g., isolated papillary muscles, atria, etc. (Lumley et al., 1977; Uhl et al., 2015; Wallis et al., 2015)], are assessed while being exposed to a given test agent. In chronically instrumented, conscious dogs, the maximal rate of pressure increase during systole in the left ventricle (LVdP/dt_max_) proved to be a sensitive and reproducible endpoint across laboratories for detecting the effects of known positive and negative inotropic agents (Guth et al., 2015). It was therefore recommended that this assessment be included in the safety pharmacological profile of all new drugs.

Despite its proven value for the demonstration of drug safety in preclinical testing, the use of LVdP/dt_max_ in animal models is usually done when the final selection of a development candidate is being made. This is a consequence of the fact that certain information needs to be available to perform such an *in vivo* study, and an adequate amount of the test article needs to be synthesized to conduct an *in vivo* study in conscious dogs, pigs, or nonhuman primates. For instance, a safety assessment is typically based on an estimation of the human dose and multiples of the predicted systemic drug concentration. Furthermore, the pharmacokinetic profile of a new compound needs to be defined to allow for an appropriate dosing to cover adequate multiples of therapeutically relevant drug concentrations. It is not uncommon to require significant compound amounts (10–20 g) to perform such a study, and this is often not available until rather late in the research phase of drug development. Furthermore, *in vivo* studies are resource-intensive, and there is a practical limitation to the number of compounds that can be tested in such studies. Also, pilot toxicity studies may be completed prior to these more involved safety pharmacology studies to identify major liabilities that may preclude further development. However, given its potential impact on drug candidate selection and overall drug safety of a new drug, it is highly desirable to have an earlier assessment of a large number of early drug candidates for potential effects on myocardial contractility, preferably in a less resource-intensive, higher throughput model.

Current approaches for assessing drug effects on cardiac contractility have at least three opportunities for refinement: **1)** ability to screen new compounds before they would typically get to animal studies (in consideration of 3R aspects in drug development), **2)** to evaluate contractile changes in ***human-derived*** test systems, and **3)** to provide mechanistic insights into cellular processes responsible for the contractile effects. The ability to detect safety relevant effects early in drug research using *in vitro* models should reduce the number of compounds advancing to *in vivo* studies. This in turn should result in reduction in the number of animal studies required to test for a given drug-induced adverse effect. Advances in technology are providing opportunities to design *in vitro* test systems capable of detecting drug-induced effects on the myocardial inotropic state. This would offer substantial advantages for drug testing. An *in vitro* system would provide higher capacity, have faster turnaround times, and have substantially lower compound requirements than *in vivo* studies. This would make such assessments feasible earlier in the drug research timeline and thereby allow screening of chemical structures early in development for unwanted inotropic activity. Furthermore, if the model was based on human cells (primary or derived from human stem cells), there should be fewer questions concerning the translation of such results to the human, as is the case when using data from animal-based studies. Finally, the system could also allow for concurrent evaluation of biochemical endpoints and of cellular gene and protein expression to facilitate mechanistic insight. Evaluation of possible biomarkers in the cell media that may support use of novel or emerging circulating safety biomarkers and interpretation relative to response signatures of known toxicants will also help to understand the potential clinical manifestation.

There is currently a large amount of activity being devoted to the development of *in vitro* testing systems for detecting inotropic activity (Godier-Furnemont et al., 2015; Wallis et al., 2015; Del Alamo et al., 2016; Mannhardt et al., 2016; Lee et al., 2017; Lemoine et al., 2017; Ribeiro et al., 2017; Sala et al., 2018). A prospective description of the needed characteristics of such a test system would help guide the design of these systems. It must also be considered for which purpose a test system will be employed; therefore, we propose three basic potential uses of these systems. First, there is a need for systems suitable for early drug screening and prioritization amongst multiple drug candidates. Second, there may also be a potential role in supporting a regulatory safety package, analogous to the approach being used in the CiPA (Comprehensive *in vitro* Proarrhythmia Assay) initiative (Fermini et al., 2016; Vicente et al., 2018). Finally, the test system could be useful for determining the mechanisms responsible for effects seen clinically and to identify or characterize new or emerging circulating safety biomarkers. Therefore, prospective considerations for a given assay should reflect its intended use. Although the ultimate goal would be a single model system to cover all three use cases, it is likely that multiple models may be required to cover all possible uses.

## Considerations in the Design of an *In Vitro* Test System

### Performance Standards

The contractile force of the myocardium is determined by the level of intracellular ionic calcium (basal and/or transient) and is tightly regulated by three intrinsic mechanisms in response to changes in 1) preload or sarcomere length (Frank–Starling law), 2) heart rate (Bowditch effect), and 3) neurohumoral activity (autonomic and endocrine control) (Katz, 1988; Penefsky, 1994; Endoh, 2004; Shiels and White, 2008; Gordan et al., 2015). Validation of a testing system would aim to demonstrate that these regulatory mechanisms are well-preserved and functionally resemble performance of the myocardium *in vivo*. Hence, a positive correlation between length-tension or between force-frequency and an increase in contractile force by sympathetic activation or elevated intracellular calcium concentration should be demonstrable. A direct measurement of isometric force (against afterload) is preferable as an endpoint with derivative outputs such as end-relaxation (diastolic) tension, peak-contraction (systolic) tension, velocity, and duration of contraction or relaxation, etc. to analyze a full cycle of contraction and relaxation. However, surrogate measures may be used, especially in a high-throughput assay setting, as long as the physiological relevance, power of accuracy, and possible limitations of the surrogate measurement are well defined.

**Figure 2:**
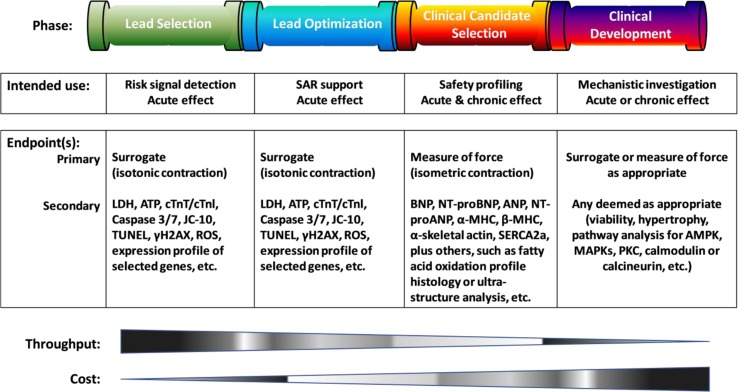
A schematic overview of endpoints proposed to use at different phases of drug discovery and development, along with the required throughput and associated cost. The primary endpoint refers to functional measurements to quantify contractile force generation indirectly with a surrogate (often employing optical technique to detect shortening—namely, isotonic contraction of cardiomyocytes beating freely without a load) or directly in the presence of both pre- and after-loads. The secondary endpoint is used to detect cell injury, potentials, or underlying mechanisms for long-term modeling. SAR, structure-activity relationship; LDH, lactate dehydrogenase; ATP, adenosine triphosphate; cTnT/cTnI, cardiac troponin T/I; TUNEL, terminal deoxynucleotidyl transferase (TdT) dUTP nick-end labeling; γH2AX, gamma H2A histone family member X; ROS, reactive oxygen species; BNP, B-type natriuretic peptide; ANP, atrial natriuretic peptide; NT-proBNP or proANP, N-terminal pro B-type or atrial natriuretic peptide; α-/β-MHC, alpha- or beta-myosin heavy chain; SERCA2a, sarcoplasmic reticulum Ca2^+^ ATPase; AMPK, 5’AMP-activated protein kinase; MAPKs, mitogen-activated protein kinase; PKC, protein kinase C.

Characterization of cellular preparations and scientific qualification of analytic platforms may be undertaken with physiological and/or pharmacological interventions as listed in **Table 1**. The validation study design, i.e., selection of reference compounds, endpoints, or exposure/observation period, is expected to be tailored to fit the purpose of its specific use. A simple testing system using surrogate endpoints (**Figure 2**) may well serve the screening strategy to support risk identification or lead optimization efforts. A more sophisticated testing system capable of assessing the length-tension, force-frequency relationship, neurohumoral influence, and calcium reliance would be highly desirable for comprehensive safety profiling of clinical candidates or for aiding in mechanistic investigation to support regulatory filing and clinical development. Of note, testing systems developed so far are primarily focused on the assessment of acute effects (with ≤1-h drug exposure). This is suitable for a screening model, but longer drug exposure (≥3 days) for the analysis of chronic effects may be desirable since it is well-known that certain drug classes, e.g., chemotherapeutics, cause delayed contractile deficiency as a result of myocardial remodeling or cardiomyopathy (Mellor et al., 2011; Senkus and Jassem, 2011; Curigliano et al., 2016).

**Table 1:** List of compounds or interventions that would be useful in profiling the performance of a novel *in vitro* testing system due to the involvement of various inotropic mechanisms.

Intervention	Mode of action	Acute inotropic effect	Dose range	Note	References of studies with human myocardia or stem cell–derived cardiomyocytes
Stretch	Frank–Starling Law of Heart	Positive	Stepwise (∼50 µm/step)	Until a plateau is achieved	(Tulloch et al., 2011; Iribe et al., 2014; Mannhardt et al., 2016; Ruan et al., 2016)
Pacing	Force-frequency relationship	Positive	0.5–6 Hz	Until a plateau is achieved	(Brixius et al., 2002; Godier-Furnemont et al., 2015; Mannhardt et al., 2016; Tiburcy et al., 2017)
Extracellular [Ca2+]	Excitation-contraction coupler	Positive	0.1 to 5 mM	Until a plateau is achieved	(Nabauer et al., 1988; Lenz et al., 1995; Stoehr et al., 2014; Godier-Furnemont et al., 2015; Mannhardt et al., 2016; Ruan et al., 2016; Mannhardt et al., 2017; Tiburcy et al., 2017)
Hypoxia	ATP depletion	Negative	≤1% O_2_ up to 12 h	HIF-1α* and cell injury markers	(Hafez et al., 2018; Hidalgo et al., 2018)
Isoprenaline	β-Adrenergic agonist	Positive	0.001–1 µM		(Nabauer et al., 1988; Lenz et al., 1995; Brixius et al., 2002; Hayakawa et al., 2014; Stoehr et al., 2014; Kijlstra et al., 2015; Mannhardt et al., 2016; Mannhardt et al., 2017; Tiburcy et al., 2017)
Norepinephrine	α-Adrenergic agonist	Positive	0.001 to 10 µM	Negative (chronic effect)	(Liu et al., 2012; Tiburcy et al., 2017)
Carbachol	Cholinergic agonist	Negative	1, 10 µM	Following β-adrenergic activation	(Jakob et al., 1989; Stoehr et al., 2014; Mannhardt et al., 2016)
Digoxin	Na+/K+-ATPase inhibitor	Positive	0.001–2 µM		(Nabauer et al., 1988; Guo et al., 2011a; Mannhardt et al., 2017)
Uabain	Na+/K+-ATPase inhibitor	Positive	0.03–3 µM		(Nabauer et al., 1988; Guo et al., 2011a; Mannhardt et al., 2017)
Bay K-8644	L-Ca2+ channel opener	Positive	0.01–0.3 µM		(Gossmann et al., 2016; Mannhardt et al., 2016)
Nifedipine	L-Ca2+ channel blocker	Negative	0.003–3 µM		(Guo et al., 2011b; Mannhardt et al., 2017)
Verapamil	L-Ca2+/hERG channel blocker	Negative	0.03–0.3 µM		(Hayakawa et al., 2014; Kijlstra et al., 2015; Gossmann et al., 2016)
Caffeine	RyR2 activator	Positive	100–10,000 µM		(Greensmith et al., 2014; Kim et al., 2015; Ribeiro et al., 2017)
Ryanodine	RyR2 inhibitor	Negative	0.1–10 µM		(Kirby et al., 1992; Godier-Furnemont et al., 2015; Mannhardt et al., 2016; Mannhardt et al., 2017)
Thapsigargin	SERCA2a inhibitor	Negative	0.01–1 µM		(Kirby et al., 1992; Godier-Furnemont et al., 2015)
EMD-57033	Myosin ATPase activator	Positive	0.1–10 µM		(Mannhardt et al., 2016)
Blebbistatin	Myosin II ATPase inhibitor	Negative	0.001–1 µM		(Skwarek-Maruszewska et al., 2009; Guo et al., 2015; Obergrussberger et al., 2016)
Milrinone	PDE inhibitor	Positive	0.03–300 µM		(Lenz et al., 1995; Mannhardt et al., 2017)
Forskolin	Adenylyl cyclase activator	Positive	0.3–10 µM	More effective on failing hearts	(Nabauer et al., 1988)
dB-cAMP	Member permeable cAMP	Positive	30–3,000 µM	More effective on failing hearts	(Nabauer et al., 1988)
Histamine	H-receptor agonist	Positive	0.3–100 uM		(Nabauer et al., 1988; Khankoeva et al., 1997)
Angiotensin II	Ang II-receptor agonist	Positive	0.001–0.5 µM	Negative (chronic effect)	(Lenz et al., 1995; Horton et al., 2016)
Endothelin-1	ET-1 receptor agonist	Positive	0.0003–0.1 µM	Negative (chronic effect)	(Lenz et al., 1995; Meyer et al., 1996; Grosberg et al., 2012; Horton et al., 2016)
SEA0400	NCX inhibitor	Positive	0.01–1 µM		(Bolck et al., 2004; Mannhardt et al., 2016)

### Cell Source, Species, and Phenotype

Human cells as a primary source material for an *in vitro* system optimized for relevance to patients should be a priority. Rapid advances in the ability to promote specific cell lineage differentiation from human-induced pluripotential stem cells (hiPSC), which themselves have been de-differentiated from human cells as easily accessed as fibroblasts, have facilitated the development of *in vitro* systems utilizing human or even patient-specific cells (Karakikes et al., 2015). Cardiomyocytes are a cell type for which this has been particularly successful. However, challenges have been experienced in defining and fully replicating a mature, adult phenotype (Denning et al., 2016). To be relevant, cardiomyocytes *in vitro* should express the structural and functional features of cardiomyocytes *in vivo*. These include cytoskeletal elements like actin and myosin myofibrils, regulatory complexes like tropomyosin and its attendant troponins, calcium channels like L-type, ryanodine, and SERCA as well as ATP-producing mitochondria. The energetics/metabolism of the cell should also be relevant to the *in vivo* setting, recognizing that adult cardiomyocytes predominately use free fatty acids as a primary substrate, but have the ability to shift to a more glycolytic pathway in times of oxygen limitation (ischemia) or restricted lipid availability. For example, a cardiomyocyte completely dependent on glucose for ATP production likely has a very different susceptibility to injury than one using lipid-dependent energy production. An *in vitro* system intended to model the *in vivo* physiology of cardiomyocyte contractility should be cyclically stimulated to contract and should be under modifiable stretch-related stress. Optimally, the system should also include an ability to measure the force of contraction and acute, drug-induced changes as well as potential changes in cell length and contractile performance with longer drug exposure. The differences between primary cardiomyocytes and hiPSC-derived cardiomyocytes are summarized in **Table 2**. Different models may be required for these various measurements.

**Table 2:** Comparison between primary human ventricular cardiomyocytes and ihPSC-derived cardiomyocytes.

	Characteristic	Primary cardiomyocyte	hiPSC cardiomyocyte
Morphology	Shape	Rod	Round
	Nucleation	Mostly tetraploidy	No polyploidism
	Alignment	Longitudinal	None
	Myofibril orientation	Longitudinal	None
	Sarcomere banding	Z, I, A, H, and M bands	Z and I bands only
	T-tubule	Present with Z-disks	Not present
Contractility	Adrenoreceptors	ß1-dependent	ß2-dependent
	Contractile proteins	TNNI1, MYH6, MLC2a, N2B	TNNI3, MYH7, MLC2v, N2BA
	Contractile force	10–50 mN/mm^2^	0.1–0.5 mN/mm^2^
	Sarcoplasmic reticulum	Well developed;	Low expression of
		High expression of RYR2 and SERCA2a	RYR2 and SERCA2a
Metabolism	Substrate	Fatty acid	Glucose and lactate
	Mitochondria	High numbers	Low numbers

Adapted from Tan and Ye. J. of Cardiovasc. Trans. Res (2018) 11:375–392 (Tan and Ye, 2018).

There are now *in vitro* test systems available that appear to successfully induce a shift to a phenotype more closely resembling the phenotype of a primary cardiomyocyte. In general, this entails the use of hiPSC cardiomyoctes in artificial three-dimensional tissues that can then be placed under mechanical stress. Over time, there are multiple changes of the cell structure and orientation, as well as intracellular modifications which are more reminiscent of primary cardiomyocytes (Tiburcy et al., 2017; Zhao et al., 2019).

### Analytical Features

*In vivo* biology is complex, and the contracting heart with its interdependence on endocrine and autonomic signaling, oxygen, and other energy substrate supplies, as well as fluid volumes and pressures in the vascular compartment, is no exception. The presence or absence of all of those interdependencies will influence the level at which an *in vitro* system (even one populated by human cells) will represent human *in vivo* physiology and pathophysiology. Alternatively, the analytical complexity of a testing platform affects its physical footprint, reproducibility, skills required to use it, cost, and its throughput. An *in vitro* system for testing inotropy should have the complexity necessary for the specific context of its intended use but provide the throughput necessary to support the stage of drug development in which it is to be used.

Assessment of inotropic activity requires use of functional endpoints to be monitored with a non-invasive technique under a physiological environment. For the detection of drug effects on contractility, primary and preferably simple endpoints may be advantageous. However, systems that also allow for the evaluation of additional (secondary) endpoints are attractive, as they would allow one to investigate mechanisms underlying observed changes in inotropic activity or to detect induction of long-term chronic remodeling such as pathological hypertrophy and cardiomyopathy (Horton et al., 2016; Maillet et al., 2016; Tiburcy et al., 2017). As illustrated in **Figure 2**, selection of endpoints, acute (≤1 h post-dose), or subacute (≥3 days post-dose) effects will be based on the purpose of use.

It is also important to consider the quantitative endpoints that will provide useful insights into the physiologic health of the cellular system (particularly as it relates to inotropic function) and relevance to the *in vivo* condition. Cardiac inotropic cellular systems should have some mechanism for measuring cellular movement. Most of the systems that are currently available for drug testing employ surrogate endpoints to analyze isotonic contractile force generation indirectly by measuring shortening/movement with optical technology (Grosberg et al., 2012; Hayakawa et al., 2014; Stoehr et al., 2014; Huebsch et al., 2015; Kijlstra et al., 2015; Pointon et al., 2015; Mannhardt et al., 2017; Pointon et al., 2017) or with electrical impedance (Guo et al., 2011a; Doerr et al., 2015) in cardiomyocytes beating spontaneously in the absence of preload and afterload. Of note, rhythmic changes in impedance of beating cardiomyocytes correlate well with field potentials measured simultaneously during a contraction and relaxation cycle and are sensitive to myosin II ATPase inhibition (Doerr et al., 2015; Guo et al., 2015), suggesting impedance to be a physiologically relevant surrogate of myocyte contraction. Nevertheless, additional studies are needed to further define the relationship of impedance to contraction *per se*. Contraction and relaxation times may also be useful parameters representing duration of *in vivo* systole and diastole, respectively. Given that cardiomyocytes *in vivo* are under constant and varying stretch stress against which force is generated, a system relevant to the *in vivo* setting is expected to have the ability to mimic and measure those features. Lastly, calcium concentrations and movement within cardiomyocytes may also be useful as they are important regulators of the contractile machinery. Several established systems measure “calcium sparks” or transients using voltage-sensitive dyes (Qian and Guo, 2010; Cerignoli et al., 2012; Stoehr et al., 2014; Huebsch et al., 2015; Kijlstra et al., 2015; Lu et al., 2015; Pointon et al., 2015; Dempsey et al., 2016; Hansen et al., 2017; Sirenko et al., 2017).

Aside from endpoints that are most directly reflective of acute changes in inotropic activity (e.g., cell movement, contractile force development, calcium cycling), there are secondary endpoints that might also be useful to measure as key physiologic enablers. Cell health and viability will impact the force generation ability of a cellular system whether the primary injury is in the inotropic machinery or not. Cell viability assays have become a standard tool used in drug research and development (Stoddart, 2011). Measurement of lactate dehydrogenase (LDH) release and/or caspase 3/7 activation provides a simple, fast, and cost-effective method to monitor membrane permeabilization and apoptotic cell death, respectively, and can be used to detect degenerative effects on cardiomyocytes in a culture system containing a pure cardiomyocyte population (Guo et al., 2015). However, release of troponins would be more relevant to cardiomyocytes when they are in a multi-cellular culture system as it is measured *in vivo* as a biomarker of cardiomyocyte injury and is a component of the contractile regulatory complex (O’Brien, 2008). Contraction and relaxation are both energy-requiring activities dependent on an intact ATP-generating mitochondrial machinery. Accordingly, measures of mitochondrial function and ATP production may be useful (Rana et al., 2012; Guo et al., 2015). Lastly, gene or protein expression is often used as a reflection of normal or abnormal cellular responses. It is known that pathological hypertrophy is associated with an increase in expression of atrial natriuretic peptide (ANP), B-type natriuretic peptide (BNP), β-myosin heavy chain (β-MHC), and α-skeletal actin and a decrease in expression of α-MHC and Ca2+ uptake pump (sarcoplasmic reticulum Ca2+ ATPase, SERCA) or in fatty acid oxidation metabolism *via* modulation of several signaling pathways including protein kinase C (PKC), 5’AMP-activated protein kinase (AMPK), and mitogen-activated protein kinase (MAPKs) (Barry et al., 2008; Bernardo et al., 2010; Schirone et al., 2017). Acute changes in gene expression are sometimes used to provide insights into chronic outcomes. For example, short-term exposure to a compound that induces cardiomyocyte hypertrophy might elicit upregulation of actin and myosin genes before changes in cell size are identifiable.

As noted earlier, throughput capabilities are significantly influenced by the biological and analytical complexity of an *in vitro* system. A system that included all the analytical features noted above would likely struggle to be represented in a 96-well template at a size that would fit into a traditional laboratory incubator. Accordingly, trade-offs are likely to be made dependent upon the intended use of the testing system.

### Testing Strategies and Their Validation

Confidence that a novel *in vitro* system will meaningfully support decisions to progress candidate drugs comes from evidence and experience. A novel *in vitro* system must have a clear line of sight to a specific use that could be articulated using standards defined by a paper like this one. The system should have some demonstrated ability to be reproducible and perform within accepted analytical limits, particularly for primary endpoints. This performance is generally considered “validation” and could involve demonstrating reproducibility over individual runs, over time, and between laboratories. Assay sensitivity needs to be reproducible and documented. Validation of platforms and assays for measuring cardiac contractility *in vitro* involves the use of different compounds with known clinical effects that induce direct contractile changes of cardiomyocytes. One such list is presented in **Table 1**, but various published studies already report compounds used in contractile assays (Sirenko et al., 2013; Adolph et al., 2015; Butler et al., 2015; Jacob et al., 2016; Afik et al., 2017; Pointon et al., 2017). However, there are no universally accepted lists to assay all aspects of cardiac contractility. In fact, defining such lists in the context of a contractility study involves careful consideration of the desired mechanistic deliverables and the motivating scientific questions of such a study. Assaying for different mechanisms of cardiotoxicity is a good example of how lists of compounds may vary to fit the context of use of *in vitro* assays (Baan et al., 2015). Such compounds that normally affect cell contractility through specific mechanisms of action and compound-induced contractile variations observed experimentally can be informative about the activity of molecular pathways that regulate contractility. This experimental information on contractile physiology is particularly useful when using iPSC-cardiomyocytes because these cellular models are often found to lack mature contractile properties and may therefore not represent drug-induced changes seen with ventricular cardiomyocytes. In such cases, making decisions on the study outcome may not be warranted (Sirenko et al., 2013; Butler et al., 2015; Guth et al., 2015; Varga et al., 2015; Jung et al., 2016; Mannhardt et al., 2016; Lee et al., 2017; Pointon et al., 2017).

Translation of the assay effects to human outcomes is another challenge that needs to be addressed. The promise associated with the use of specifically human-induced stem cell–derived cardiomyocytes is that they will provide an enhanced translation on the basis of the human genetic background. However, if the currently available stem-cell-derived cardiomyocytes have not succeeded in replicating the phenotype of the human ventricular cardiomyocyte, which thought to be the drug target in this context, then a translation cannot be assumed. It is common to characterize assays using drugs with known effects in patients. This is certainly a useful approach, as was used in the characterization of *in vivo* preclinical studies to detect effects on myocardial contractility (Guth et al., 2015). However, that approach as a sole means of assay validation is challenged in many ways. Many of the tool compounds used for such testing are drugs that were designed to target the heart by known mechanisms, so are useful for “mechanistic characterization.” In contrast, the drugs that have the most concerning cardiac inotropic effects are those not intended to target the heart. Many of these compounds are removed from development before they are given to patients (i.e., the clinical outcome is unknown) or are associated with cardiac contractile deficiency in small numbers of patients over longer periods of dosing by mechanisms that are largely unknown. Also, the biological targets of pharmacology are constantly changing as we learn more about biology and search for drugs that more effectively treat more chronic and complex diseases. Accordingly, it is impossible to qualify a test system fully for mechanisms yet unseen.

The challenges above dictate that qualification and confidence in a novel test system are often an evolutionary process that begins with alignment of the system to defined standards, validating its reproducibility, performing some level of mechanistic qualification with well-characterized compounds, and then using the system in real drug development efforts to build confidence in its value. For cardiac contractility, a system that could be used to screen novel compounds prior to animal studies and/or to investigate mechanisms of inotropic effects for drugs that have been studied in animals or, better yet, patients would be a relevant starting point in this evolution. Whereas there seems to be much room for further improvements in the currently available test systems, the continued research in this area, the substantial progress made to date, and the clear need for such test systems would indicate that the goals may be attainable in the near future.

### *In Vitro* System Considerations in the Context of Its Intended use

There was a time when costs for a novel system for which there was confidence of value in drug development were less important than they are today. However, the pharmaceutical industry is experiencing unprecedented economic challenges, such that cost considerations have become very important. The confidence and evidence of value that are required to adopt a novel test method; the magnitude of that value and even the willingness to explore unproven innovation are all affected by these challenges. Novel test systems must consider economic value and find an acceptable balance between cost and impact for the specific use intended. That balance varies across those contexts.

### Considerations for an Inotropic Screening Assay

The early detection of drug-induced effects on the inotropic state of the myocardium (= cardiac contractility) is desirable to identify risk early and to guide further chemical optimization and the ultimate progression decisions for drug candidates. Early screening assays may also utilize surrogate endpoints of inotropy if they facilitate throughput (**Figure 2**). Obviously, this would not apply to projects targeting modulation of the inotropic state of the heart as part of its therapeutic efficacy. Two key hallmarks of a successful screening assay are having an adequate throughput and acceptable costs, which allow for the testing of large numbers of compounds. It may be assumed that compounds entering a screening assay of inotropic activity will have already demonstrated activity on its target. Thus, rather than considering thousands of compounds for such screening, an assay should be able to test numbers ranging from the tens to hundreds of compounds within a limited (days to weeks) period of time.

The costs for running such a screening assay must also be appropriate for the number of compounds to be screened. Since this could potentially include up to hundreds of compounds per year/project, the unit price cannot be too high. For instance, if a given assay includes the assessment in duplicate of five different test concentrations, testing of a single compound would require 10 measurements. An assay with a cost per sample of 100 US dollars would then have a price per compound of 1,000 dollars. Lower costs per data point would encourage a higher usage of any test system.

For a given assay system, the key output parameters should be clearly identified and the system should be characterized such that the sensitivity and selectivity for changes in those parameters are defined. For a screening assay, one is usually willing to accept a certain amount of testing error. Tolerance for false negative results is limited. Keeping false-positive results to a minimum is desirable, as this would deprioritize compounds that may otherwise be attractive drug candidates.

An important consideration, even for use in a screening assay, is the assessment of effect in terms of a concentration-dependent response. Thus, some estimate of the therapeutic concentration of a drug *versus* that concentration first associated with an undesirable action on myocardial contractility is desirable. However, it must be recognized that in early stages of drug discovery, the concentrations of a drug leading to therapeutic activity may be poorly defined, in which case a wide range of concentrations should be covered to ensure adequate coverage of the target and interrogation of potential toxicity.

Finally, it would be preferred that even a screening assay has an endpoint that is mechanistically related to the physiology of myocardial contraction, but for this purpose, i.e., for screening, it is not an absolute requirement. Ideally, the endpoint would relate to, or predict a change in function in sarcomere shortening or relaxation against a load, as one would see in terms of tension or pressure development and the actual shortening or relaxation of the sarcomere or muscle. As seen in **Figure 1**, there is an intrinsic role of intracellular calcium transient for both the sarcomere activation and relaxation, such that an assessment of the calcium-transient could prove useful as a surrogate endpoint. Whereas that is certainly of interest and could serve as an experimental endpoint, one must also be cautioned with regard to potential uncoupling of calcium flux to actual shortening, relaxation, or tension development (Fan et al., 1997; Ter Keurs et al., 2006; Eisner et al., 2017).

### Considerations for an *In Vitro* Assay for Examining the Mechanism of Inotropic Modulation

Once a given compound has been shown to have effect on myocardial contractility, there may be interest in understanding the mechanism responsible for the effects observed. As seen in **Figure 1**, the current understanding of electromechanical coupling suggests a variety of potential drug targets that could modulate the overall process and lead to an inotropic effect. One of the current challenges facing the use of stem cell–derived cardiomyocytes for testing drug for inotropic effects has been that cells from different vendors, or cells derived using various protocols, have been shown to have different physiological phenotypes with regard to these intracellular processes (Kane et al., 2015; Denning et al., 2016; Gadeberg et al., 2016). Whereas this may limit the use of some cells for general screening use, once it is understood how they differ from native human cardiomyocytes, some of these cell types could prove very useful for mechanistic studies. This will require a thorough understanding of the intracellular components in each of the cell types, information which is not currently available, but does offer the potential for detailed mechanistic studies in the future. Once a given cell type (i.e., cell coming from a particular vendor or derived from stem cells using a well-defined protocol) has been adequately characterized, its use for a particular type of study can be optimized according to these physiological and pharmacological characteristics. Furthermore, use of genetically modified cells may eventually allow assays to provide a mechanistic insight to drug-induced effects

### Requirements for an *In Vitro* Assay With Regulatory Status

With the CiPA initiative as a template, one might anticipate the role of a well validated *in vitro* assay for potential inotropic effects in the regulatory safety assessment of a new drug. If used for this purpose, a negative outcome (no effect on inotropic state) would need to be a very robust result with a very low incidence of false negative outcomes. The requirement for high assay sensitivity may require a different model in comparison to one designed for screening that requires high specificity. Overall, there is a stronger rationale for the use of human primary tissue or human-induced stem cell–derived cardiomyocytes to avoid potential issues of inter-species differences in translation. On the other hand, for regulatory purposes, far fewer compounds will receive such testing such that both the throughput and costs associated with the testing become less critical. If this were to be included into the safety pharmacology core battery, this type of study would be reserved only for compounds being positioned to enter first-in-man clinical trials. To be a requirement of a regulatory package, the assay would be expected to have defined benefits in its ability to translate to man, and thereby further support, an *in vivo* preclinical CV study in which effects on cardiac contractility are assessed.

## Conclusion

The detection and avoidance of unwanted drug-induced effects on cardiac contractility have become of increasing importance in the search for novel and safe drugs (Guth et al., 2015). The development and use of practical, efficient, and appropriate *in vitro* test systems will be a valuable early addition to strategies that identify the best possible drug development candidates. The characteristics of a successful cell or tissue-based testing platform for cardiac contractility will be dictated by its intended use. In this article, general considerations for *in vitro* test systems have been presented with the intent of guiding the development of new testing platforms that will find utility in drug research and development strategies. In this way, further development activities and refinements can be aimed at real-world needs and applications.

## Author Contributions

All authors contributed various components of the manuscript. Specifically, BB provided the concept and BG wrote the first draft. All authors also contributed to manuscript revision, read and approved the submitted version.

## Funding

The writing of this manuscript has been funded in part with federal funds from the National Cancer Institute, National Institutes of Health, under contract no. HHSN261200800001E.

## Disclaimer

The content of this publication does not necessarily reflect the views or policies of the Department of Health and Human Services, including the FDA, nor does mention of trade names, commercial products, or organizations imply endorsement by the U.S. Government.

## Conflict of Interest Statement

BG was employed by Boehringer-Ingelheim. ME was employed by Amgen. CF and GG were employed by AbbVie. LG was employed by Leidos Biomedical Research. TZ was employed by Genentech. KC was employed by GlaxoSmithKline.

The remaining authors declare that the research was conducted in the absence of any commercial or financial relationships that could be construed as a potential conflict of interest.
